# Decreased expression and clinical significance of miR-148a in hepatocellular carcinoma tissues

**DOI:** 10.1186/s40001-014-0068-2

**Published:** 2014-12-02

**Authors:** Linjiang Pan, Suning Huang, Rongquan He, Minhua Rong, Yiwu Dang, Gang Chen

**Affiliations:** Department of Radiotherapy, First Affiliated Hospital of Guangxi Medical University, 6 Shuangyong Road, Nanning, Guangxi Zhuang Autonomous Region 530021 PR China; Department of Medical Oncology, First Affiliated Hospital of Guangxi Medical University, 6 Shuangyong Road, Nanning, Guangxi Zhuang Autonomous Region 530021 PR China; Research Department, Affiliated Cancer Hospital, Guangxi Medical University, 71 Hedi Road, Nanning, Guangxi Zhuang Autonomous Region 530021 PR China; Department of Pathology, First Affiliated Hospital of Guangxi Medical University, 6 Shuangyong Road, Nanning, Guangxi Zhuang Autonomous Region 530021 PR China

**Keywords:** miR-148a, Hepatocellular carcinoma, Metastasis, Paraffin-embedded tissues, RT-qPCR

## Abstract

**Background:**

Aberrant expression of microRNA-148a (miR-148a) has been reported in several types of malignancies. However, its expression and clinicopathological significance in hepatocellular carcinoma (HCC) has not been entirely clarified. Our objective was to investigate the clinicopathological contribution of the miR-148a expression in HCC formalin-fixed paraffin-embedded (FFPE) tissues.

**Methods:**

Eighty-nine HCC and their para-cancerous liver tissues were recruited. Total mRNA including miRNA was isolated and miR-148a expression was determined by using real time RT-qPCR. Furthermore, the relationship between the miR-148a level and clinicopathological features was explored.

**Results:**

Significantly lower miR-148a expression in HCC tissues was observed than that in adjacent noncancerous hepatic tissues. miR-148a expression was also correlated to clinical TNM stage, metastasis, status of capsular infiltration and numbers of tumor nodes.

**Conclusions:**

Underexpression of miR-148a might be associated with HCC tumorigenesis and deterioration of HCC. miR-148a might act as a suppressor miRNA of HCC and it therefore has a potential role in prognosis of HCC patients.

## Background

Hepatocellular carcinoma (HCC) is ranked as the sixth most frequent cancer and the third leading cause of cancer-related deaths globally [1,2]. In 2012, 782,000 new cases and 746,000 deaths from HCC occurred in the whole world. The burden of the disease is the highest in Eastern Asia, sub-Saharan Africa, and Melanesia with the endemic infection of hepatitis B virus (HBV). In the meantime, in Japan, United States, and Europe, hepatitis C virus (HCV) infection is predominant, and consequently, it has become the key risk factor for contracting HCC in these regions [3–7]. Although the improvement of molecular biology has led to the identification of new tumor markers that play vital roles in the treatment and prognosis of HCC, more tumor markers are still required for effective early diagnosis and monitoring of the curative effect of HCC [8–15]. MicroRNAs, a major class of small non-coding RNAs, are well-conserved very small RNA molecules (20 to 22 nucleotides) that can negatively modulate gene expression post-transcriptionally. Accumulating evidence over the past decades highlights the significance of microRNAs as vital regulators of many essential physiological processes such as cell proliferation, differentiation, apoptosis, and embryonic development [16]. Dysregulation of microRNA expression has also been inferred in many diseases as well as in cancers [17–21]. Extensive profiling studies over recent years have shown that a variety of miRNAs are abnormally expressed in HCC [22,23]. Among all the HCC-related miRNAs, miR-148a has been reported to be significantly reduced in HCC tissues, compared with normal livers [24,25]. However, the relationship between miR-148a level and the clinicopathological parameters of HCC remains obscure. In the current study, we therefore investigated the expression of miRNA-148a in HCC and their matched adjacent noncancerous liver tissues in formalin-fixed paraffin-embedded (FFPE) surgically resected samples. Furthermore, we studied the relationship between miR-148a expression and clinicopathological characteristics of HCC.

## Methods

### Tissue samples

This retrospective study included 89 cases of HCCs and their paired paraneoplastic liver FFPE tissues. The age of the HCC patients ranged from 29 to 82 years old, with a mean age of 52 years. Clinicopathological information extracted from medical records has been summarized in Table 1. Adjacent noncancerous liver tissues were at least 2 cm away from the tumor node. All cases were initial hepatectomies without treatment and from hepatectomies performed in the First Affiliated Hospital of Guangxi Medical University, PR China between March 2010 and December 2011. The study protocol was approved by the Ethical Committee of the First Affiliated Hospital of Guangxi Medical University. Written informed consent was obtained from the patients and clinicians for the usage of the samples for research. All samples were reviewed and diagnosed by two independent pathologists.

**Table 1:** **Relationship between the expression of miR-148a and clinicopathological features in hepatocellular carcinoma (HCC)** $\left(\overline{\boldsymbol{x}}\pm \boldsymbol{s}\right)$

**Clinicopathological features**		**n**	**MiR-148a relevant expression**		
			***2*** ^**-△*cq***^	***t***	***P***
Tissue	HCC	89	0.87 ± 0.50	−7.762∆	< 0.001
	Adjacent noncancerous liver	89	1.44 ± 0.61		
Age	≥ 50	43	0.83 ± 0.49	−0.727	0.469
	< 50	46	0.91 ± 0.51		
Gender	Male	72	0.88 ± 0.50	0.392	0.696
	Female	17	0.83 ± 0.53		
Differentiation	Well	6	0.88 ± 0.40	*F* = 0.531^a^	0.590
	Moderately	57	0.91 ± 0.52		
	Poorly	26	0.79 ± 0.48		
Clinical TNM stage	I to II	19	1.17 ± 0.58	3.106	0.003
	III to IV	70	0.79 ± 0.45		
Metastasis	Yes	46	0.74 ± 0.51	−2.537	0.013
	No	43	1.00 ± 0.46		
With cirrhosis	Yes	43	0.86 ± 0.52	−0.143	0.886
	No	46	0.88 ± 0.48		
AFP (μg/L)	≥ 400	35	0.90 ± 0.51	−0.015	0.988
	< 400	38	0.90 ± 0.49		
Portal vein tumor embolus	Yes	29	0.76 ± 0.53	−1.400	0.165
	No	60	0.92 ± 0.48		
Tumor capsular infiltration	No capsular or capsular infiltration	48	0.76 ± 0.49	−2.308	0.023
	No capsular infiltration	41	1.00 ± 0.49		
Tumor nodes	Multiple	40	0.75 ± 0.47	−2.015	0.047
	Single	49	0.96 ± 0.51		
Tumor diameter (cm)	≥ 5	73	0.91 ± 0.51	1.515	0.133
	< 5	15	0.70 ± 0.46		
Vaso-invasion	Yes	32	0.79 ± 0.53	−1.116	0.267
	No	56	0.92 ± 0.49		
HBV infection	Yes	73	0.84 ± 0.51	−1.347	0.181
	No	16	1.02 ± 0.45		
HCV infection	Yes	29	0.94 ± 0.51	0.912	0.364
	No	60	0.84 ± 0.50		

∆Paired *t*-student test was performed.

^a^One-way analysis of variance (ANOVA) test was performed.

### RT-qPCR

Total RNA including miRNA was isolated from tumor sections using the miRNeasy FFPE Kit (QIAGEN, KJ Venlo, Netherlands) according to our previous reports [26–28]. RNA concentrations were determined by Nanodrop 2000 (Wilmington, DE , USA). A combination of *RUN6B* and *RUN48* was the housekeeping genes for detection of miR-148a expression [27,28]. The primers for miR-148a, *RNU6B* and *RNU48* were included in TaqMan® MicroRNA Assays (4427975, Applied Biosystems, Life Technologies Grand Island, NY, USA). The reverse primers were also used for reverse transcription with TaqMan® MicroRNA Reverse Transcription Kit (4366596, Applied Biosystems, Life Technologies Grand Island, NY, USA) in a total volume of 10 μl. Real time RT-qPCR for miRNA was performed with Applied Biosystems PCR7900. The miR-148a abundance in each sample was normalized to its references. The expression of miR-148a in the FFPE experiments was calculated with the formula 2^-Δcq^ [26–29].

### Statistical analysis

SPSS 20.0 (Munich, Germany) was performed for statistical analysis. Results were representative of three independent experiments. Values were presented as the mean ± standard deviation (SD). Student’s paired or unpaired *t*-test was used to analyze significance between paired or unpaired groups. One-way analysis of variance (ANOVA) test was used to analyze significance between groups of various differentiations. Correlations were calculated by Spearman’s method. A receiver operator characteristic curve (ROC) was employed to identify the diagnostic value. The relationship between miR-148a and recurrence was analyzed by using the Kaplan-Meier survival method. Statistical significance was determined at a *P* < 0.05 level.

## Results

**Figure 1:**
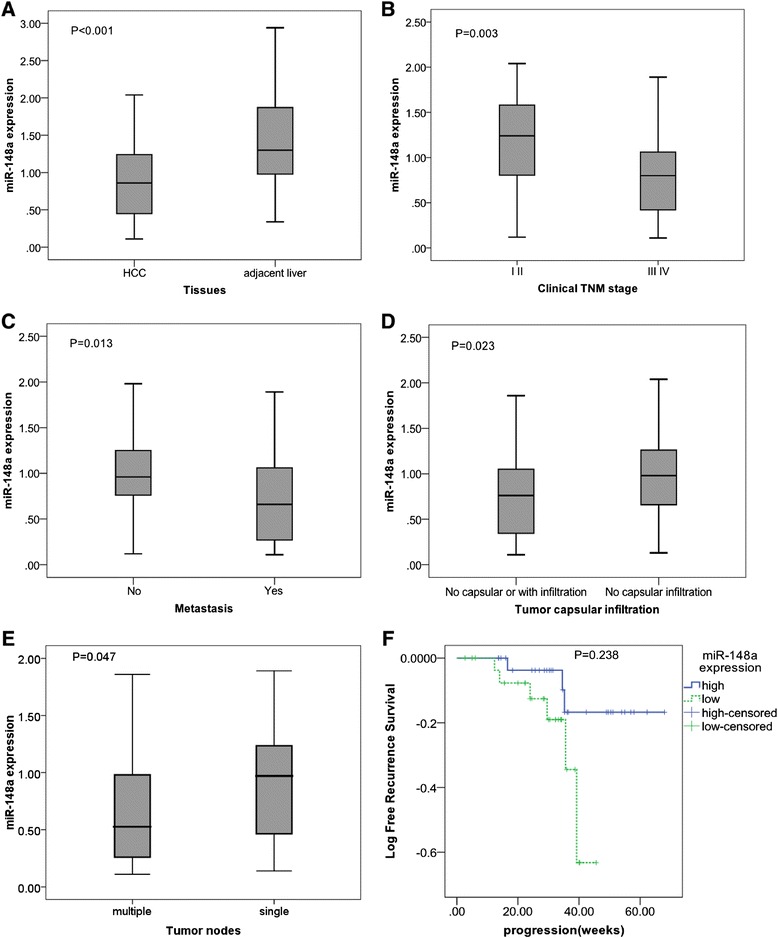
**Clinicopathological impact of miR-148a expression in hepatocellular carcinoma (HCC) tissues.** Total miRNA was extracted from HCC and their paired adjacent noncancerous liver tissues. MiR-148a expression was detected by using real time RT-qPCR and the relevant miR-148a level was calculated as compared to the reference of miR-191 and miR-103 combination. Data were shown as mean ± SD. **(A)** Different liver tissues; **(B)** Clinical TNM stages; **(C)** Metastasis; **(D)** Tumor capsular infiltration; **(E)** Tumor nodes. **(F)** The time-to-recurrence of high expression of miR-148a (higher than the median level) was 61.47 ± 3.45 months, longer than those with low expression (50.56 ± 4.15); however, the difference was not significant (*P* = 0.238).

**Figure 2:**
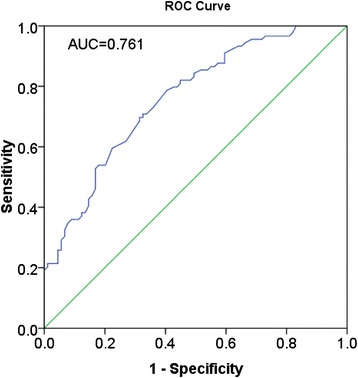
**Receiver operator characteristic (ROC) curve of miR-148a level in hepatocellular carcinoma (HCC).** The area under the curve (AUC) of miR-148a was 0.761 (95% CI 0.692 to 0.830, *P* < 0.001).

Significantly lower expression of miR-148a in the HCC tissues was detected than that in the adjacent noncancerous hepatic tissues (Table 1, Figure 1). Furthermore, the ROC curve was performed to identify the diagnostic value of miR-148a level in HCC. The area under the curve (AUC) of miR-148a was 0.761 (95% CI 0.692 to 0.830, *P* < 0.001). The cut-off value for miR-148a was the median 2^-Δcq^ 0.87. The sensitivity and specificity were 76.3% and 50.6%, respectively (Figure 2). With regard to clinical TNM stages, miR-148a expression in early stages (I and II) was remarkably higher than that in advanced stages (III and IV). Lower levels of miR-148a were found in HCC patients with metastasis, without capsular or with capsular infiltration and multiple tumor nodes, in comparison with patients of corresponding traits. In addition, according to Spearman's correlation, negative correlations were found between miR-148a expression and several clinicopathological parameters, including TNM stages, metastasis and the status of capsular infiltration. However, there was no association between miR-148a expression and other clinicopathological features, for instance, age, histological differentiation grades, cirrhosis, plasma alpha-fetoprotein (AFP) concentrations, HBV, HCV, vaso-invasion, portal vein tumor embolus or tumor size. Sixty-one among 76 patients were followed up and time-to-recurrence was collected. Time-to-recurrence for all 61 cases was 57.84 ± 3.03 weeks. The patients with high expression of miR-148a (higher than the median level) had a longer time-to-recurrence in comparison to those with low expression (61.47 ± 3.45 versus 50.56 ± 4.15), however, the difference was not significant (*P* = 0.238, Figure 1). Additionally, we performed the univariate analysis and results showed that miR-148a, as well as other parameters, was not a predictor for the recurrence of HCC in the current study (data not shown).

## Discussion

Most recently, miR-148a expression was reported by Gailhouste *et al*. to be frequently down-regulated in mouse and human HCC cell lines, as well as in biopsies of HCC patients [25]. Concurrently, consistent decreased expression of miR-148a in HCC tissues was found by Zhang *et al*. [24], as compared with normal livers. Both of the aforementioned studies used normal liver tissues as controls. Gailhouste *et al*. [25] found that miR-148a expression was reduced by more than 5-fold in HCC biopsies, relative to the normal liver group (median, 0.293 and 1.674, respectively). In the present study, accordant underexpression of miR-148a in HCC tissues was also observed, in comparison with the corresponding adjacent liver tissues in the same patients. Furthermore, the ROC curve indicates a moderate diagnostic value of miR-148a in HCC with the AUC as 0.761. Besides, lower expression of miR-148a was also detected in several HCC cell lines (HepG2, HepB3, SNU449 and SMMC7211), than the normal hepatic cell line LO2 (data not shown). The results of our study, together with those reported previously [24] [25], indicate that miR-148a plays a critical role as a tumor suppressor miRNA in hepatocarcinogenesis. However, the average fold change of miR-148a level varied in comparison to Gailhouste *et al*. [25] (1.655 versus 5.713). The different controls used by Gailhouste *et al*. [25] and our group (healthy liver tissues versus corresponding noncancerous liver tissues) may partially explain the disparity. It might be that the miR-148a expression is lower in noncancerous liver tissue of HCC patients than in liver tissue of healthy controls. It might also be of interest to investigate the dynamic change of miR-148a expression in the hepatocarcinogenesis and progression of HCC. For instance, a comparison of the miR-148a levels in normal liver, cirrhotic tissue, adjacent noncancerous liver, hepatic adenoma, atypical hyperplasia, and HCC tissues would be worthwhile exploring.

MiRNAs can be identified in serum and plasma in an extraordinarily stable form, which leads to the possibility to determine the expression of miRNAs in blood samples [30,31]. The serum level of miR-148a was detected in colorectal cancer and could be regarded as a marker to predict early tumor recurrence [32]. miR-148a was determined in the serum of other malignancies, such as breast cancer, gastric cancer and multiple myeloma [33–35]. Gailhouste *et al*. [25] also assessed the value of circulating miR-148a as a noninvasive HCC biomarker in blood serum in HCC. However, only 11 cases with HCV infection were included. The significance of circulating miR-148a in the early diagnosis and prognosis prediction of HCC remains unclarified. Further studies will be required to explore the alteration of miR-148a expression in serum and in tissue, as well as to investigate the relationship between serum miR-148a level and the clinicopathological parameters of HCC patients.

Concerning the relationship between miR-148a expression and clinicopathological parameters, Yan *et al*. [36] determined lower expression of miR-148a in 17 cases of poorly-differentiated HCC tissues relative to 15 cases of well-differentiated HCC tissues by using an miRNA microarray analysis. In the current study, similar trend was observed. In the poorly-differentiated group, the relevant miR-148a level was 0.79 ± 0.48, slightly lower than that in well-differentiated (0.88 ± 0.4) and moderately-differentiated (0.9 ± 0.52) groups. However, the differences did not reach a statistically significant level. To our knowledge, no study has reported the relationship between miR-148a expression and the clinical TNM stages of HCC. In the present study, for the first time, we have found that miR-148a expression in stages III and IV was lower than that in stages I and II. Furthermore, miR-148a expression was down-regulated in the metastatic group compared with that in the non-metastatic group. Additionally, miR-148a expression was correlated with the status of tumor cell infiltration into the capsule and the numbers of tumor nodes. The status of tumor cell infiltration and tumor nodes generally reflects tumor invasion and metastasis and disease deterioration. Zhang *et al*. [24] reported that miR-148a decreased significantly in those HCC samples with portal vein tumor thrombus. In this study, we also found that in the subgroup with portal vein tumor embolus, the miR-148a level (0.76 ± 0.53) was lower than that in the subgroup without portal vein tumor embolus (0.92 ± 0.48). Despite the fact that no statistically significant association was found between miR-148a and portal vein tumor embolus, the results from Zhang *et al*. [24] and this current study point in the same direction, that is that there is a noticeable relationship between miR-148a and the infiltration of tumor cells, migration, invasion and metastasis of HCC. Hence, it may be valuable to clinically examine miR-148a expression for the prediction of metastasis and deterioration of HCC. Next, we also investigated the relationship between miR-148a level and recurrence. The HCC patients with high expression of miR-148a had a longer time-to-recurrence than those with low expression (61.47 ± 3.45 versus 50.56 ± 4.15); however, the difference is not significant. A larger cohort is needed to determine the correlation between miR-148a and tumor recurrence in patients in the future.

The mechanisms whereby miR-148a was reduced in the advanced stages of HCC could be related to diverse target genes and pathways involved. The epithelial-mesenchymal transition (EMT) was reported to be suppressed by miR-148a via targeting Met/Snail signaling [24]. Yan *et al*. [36] further revealed that miR-148a inhibits the metastasis of HCC by blocking EMT and cancer stem cells (CSCs)-like properties through effects on the Wnt signaling. Both Gailhouste *et al*. [25] and Long *et al*. [37] discovered a possible miR-148a-DNA methyltransferase (DNMT) 1 regulatory circuit in HCC. The aforementioned target genes and relevant pathways can help to explain the role of miR-148a on the metastasis and deterioration of HCC.

## Conclusions

Together with previous reports, the current observations strongly suggest that miR-148a acts as a tumor suppressor miRNA, which plays a vital role in the tumorigenesis and deterioration of human HCC. The current finding may help to identify potential prognostic biomarkers for HCC FFPE samples and provide a promising alterative strategy for the therapeutic treatment of HCC patients.

## Abbreviations

AFP: alpha-fetoprotein
ANOVA: One-way analysis of variance
AUC: area under the curve
CSCs: cancer stem cells
DNMT: DNA methyltransferase
EMT: epithelial-mesenchymal transition
FFPE: formalin-fixed paraffin-embedded
HBV: hepatitis B virus
HCC: hepatocellular carcinoma
HCV: hepatitis C virus
miR-148a: microRNA-148a
ROC: receiver operator characteristic curve
SD: mean ± standard deviation
